# Highly sensitive wastewater surveillance of SARS-CoV-2 variants by targeted next-generation amplicon sequencing provides early warning of incursion in Victoria, Australia

**DOI:** 10.1128/aem.01497-23

**Published:** 2024-07-16

**Authors:** James E. Merrett, Monica Nolan, Leon Hartman, Nijoy John, Brianna Flynn, Louise Baker, Christelle Schang, David McCarthy, David Lister, Ngai Ning Cheng, Nick Crosbie, Rachael Poon, Aaron Jex

**Affiliations:** 1 Population Health and Immunity Division, Walter and Eliza Hall Institute of Medical Research, Parkville, Victoria, Australia; 2 Victorian Department of Health, Melbourne, Victoria, Australia; 3 Faculty of Science, The University of Melbourne, Parkville, Victoria, Australia; 4 Environmental and Public Health Microbiology Lab, Monash University, Clayton, Victoria, Australia; 5 School of Civil and Environmental Engineering, Queensland University of Technology, Brisbane, Queensland, Australia; 6 South Australian Water Corporation, Adelaide, South Australia, Australia; 7 Melbourne Water Corporation, Docklands, Victoria, Australia; Colorado School of Mines, Golden, Colorado, USA

**Keywords:** SARS-CoV-2, COVID-19, wastewater surveillance, amplicon sequencing, coronavirus, Omicron, Delta, genomic surveillance, wastewater-based epidemiology

## Abstract

The future of the COVID pandemic and its public health and societal impact will be determined by the profile and spread of emerging variants and the timely identification and response to them. Wastewater surveillance of SARS-CoV-2 has been widely adopted in many countries across the globe and has played an important role in tracking infection levels and providing useful epidemiological information that cannot be adequately captured by clinical testing alone. However, novel variants can emerge rapidly, spread globally, and markedly alter the trajectory of the pandemic, as exemplified by the Delta and Omicron variants. Most mutations linked to the emergence of new SARS-CoV-2 variants are found within variable regions of the SARS-CoV-2 Spike protein. We have developed a duplex hemi-nested PCR method that, coupled with short amplicon sequencing, allows simultaneous typing of two of the most highly variable and informative regions of the Spike gene: the N-terminal domain and the receptor binding motif. Using this method in an operationalized public health program, we identified the first known incursion of Omicron BA.1 into Victoria, Australia and demonstrated how sensitive amplicon sequencing methods can be combined with wastewater surveillance as a relatively low-cost solution for early warning of variant incursion and spread.

**IMPORTANCE:**

This study offers a rapid, cost-effective, and sensitive approach for monitoring SARS-CoV-2 variants in wastewater. The method’s flexibility permits timely modifications, enabling the integration of emerging variants and adaptations to evolving SARS-CoV-2 genetics. Of particular significance for low- and middle-income regions with limited surveillance capabilities, this technique can potentially be utilized to study a range of pathogens or viruses that possess diverse genetic sequences, similar to influenza.

## INTRODUCTION

SARS-CoV-2 (1) continues to spread globally, evolve, and mutate. These mutations, particularly within the Spike gene (2), have given rise to SARS-CoV-2 variants that are more transmissible, virulent, escape immune responses generated by prior infection or vaccination, or have developed resilience to a variety of antibody- or antiviral-driven treatments (3–5). Global genomic surveillance efforts by key consortia (6–9) have tracked the evolution of SARS-CoV-2 and classified emerging variants into lineages, sublineages, and recombinants. There have been multiple successional SARS-CoV-2 variants that have come to dominate global transmission (10). The Delta variant (B.1.617.2/AY.X) was reported in late 2020 and, within a few months, became the major SARS-CoV-2 variant in many regions (11). Omicron (B.1.1.529) emerged in late 2021, and its capacity to escape prior immunity due to marked divergence from previous SARS-CoV-2 variants facilitated its global spread within weeks (12, 13). That Omicron evolved such significant changes (14) while essentially remaining undetected, only to emerge and rapidly transform the global pandemic landscape, highlights the need for expanded viral surveillance methods.

Genomic sequencing programs have played an essential role in tracking the emergence and spread of new SARS-CoV-2 variants and remain vital to inform global, national, and local public health strategies and are also clinically relevant where variant identity can inform the course of treatment (6–9). However, genomic sequencing programs have been concentrated in high-income countries and predominantly focused on clinical samples biased toward symptomatic and hospital infections. Furthermore, as vaccination programs have expanded and levels of community infection have increased, many clinical sequencing programs have been reduced to a much smaller subset of clinical samples (3–5). Finally, a shift away from traditional PCR-based testing toward the use of at-home rapid antigen tests has significantly reduced the availability of residual PCR samples for sequencing. Combined, these compromise population representativeness and reduce the capacity to detect new viral variants as they emerge and spread.

Wastewater-based epidemiology (WBE) of SARS-CoV-2 shows high concordance with clinical genomic trends (15–20). It presents as a practical and comparatively low-cost surveillance tool, which is especially pertinent where clinical sequencing efforts are being scaled back globally or in areas where the cost of establishing a clinical whole-genome sequencing program is prohibitory. WBE can simultaneously sample many individuals in a geographical area, which supports monitoring the emergence and evolution of strains that clinical genomic sequencing might not otherwise detect(19). In Victoria, WBE is led by the Department of Health in partnership with research laboratories, water utilities, and local public health units. Over prolonged periods of low case levels in Victoria during 2020 and 2021, WBE was a critical and flexible early detection tool used to identify incursions and early outbreaks and monitor trends in SARS-CoV-2, similar to that used in many global settings (21–25). As Victoria moved from a low case-load setting into a higher community transmission context at the end of 2021, the WBE program focussed on monitoring the incursion of novel variants and the evolution of strains by looking at the diversity of non-characteristic mutations carried by variants.

**Fig 1:**
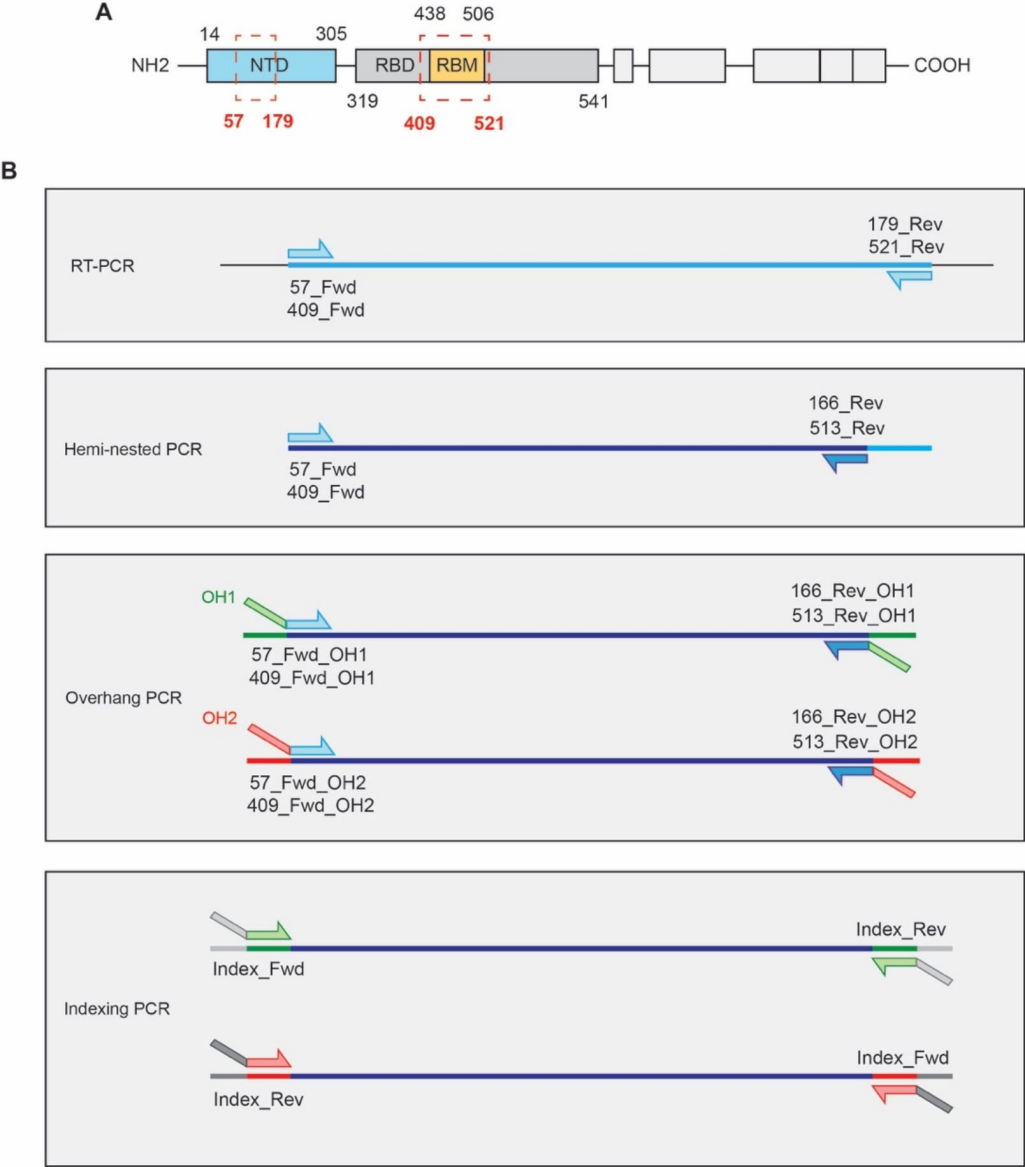
Amplicon sequencing approach. (**A**) Schematic of SARS-CoV-2 Spike protein indicating the amino acid residues that define the NTD (blue) and RBM (yellow) within the receptor-binding domain (RBD). The corresponding polymorphic regions targeted for amplicon sequencing are depicted in red. (**B**) Overview of the amplicon sequencing workflow. RNA is reverse-transcribed by RT-PCR and then further amplified by hemi-nested PCR using nested reverse primers. Sequence overhangs are added by PCR using two primer sets (OH1 and OH2). Due to the reverse orientation of OH1 and OH2, a forward index is added at the 5′ end of the OH1 and 3′ end of the OH2 amplicons. Amplicons are sequenced using 281 and 21 bp paired-end read lengths, resulting in complete sequence coverage. The primers used at each step are indicated.

Given the relatively low case-load context in Victoria, we developed a sensitive (hemi-nested) PCR method, coupled with short amplicon sequencing, which targeted two of the most variable and informative regions of the Spike gene: the N-terminal domain (NTD) and receptor binding motif (RBM) (Fig. 1). Mutations in the NTD and RBM are associated with increased transmissibility (4, 10, 26), antibody escape (26, 27), or immune evasion (28), and the unique mutation signatures can be used to type variants with a high degree of confidence. We deployed this method to type wastewater samples collected from early November to late December 2021, which coincided with higher caseloads and the emergence of the Omicron variant.

## MATERIALS AND METHODS

### Wastewater sampling

Passive wastewater sampling devices (29) were deployed at wastewater treatment plant influents (prior to treatment) and maintenance holes across Victoria and the Melbourne metropolitan sewage network with typical continuous sampling durations of 1–4 days, providing high population and geographic coverage. Localized surveillance was also conducted at Melbourne’s two major airports, which included effluent discharge from planes and wastewater from the airport. Sample frequency was purposive and varied from daily (at airports) to one to three times per week, depending on location and risk assessment. Samples reported here were retrieved from 15 November through 21 December 2021.

### RNA extraction and RT-qPCR detection of SARS-CoV-2

All samples were collected, stored, and processed for the enrichment of SARS-CoV-2 RNA, as previously described (29), however, using the MagMax Microbiome Ultra Nucleic Acid Isolation kit (ThermoScientific, USA) on a KingFisher Apex instrument (ThermoScientific, USA) per the manufacturer’s instructions. Briefly, upon retrieval of the passive sampling units, visible debris were removed and units were disassembled, yielding as many as nine passive samplers per site, per day. Electronegative membranes and cotton buds from the units were immediately used for RNA extraction or stored at −80°C until extraction. Gauzes were either frozen at −80°C or immediately eluted in a stomacher bag containing sterile 1× phosphate buffer (10 mL) mixed with Tween 80 (0.05%) and Y-30 antifoam emulsion (0.001%). After stomaching at 200 rpm for 2 min, the elution buffer was filtered through an electronegative membrane (47 mm, 0.45 µm pore size) and used immediately for RNA extraction as per reference (29).

Each RNA extract was first tested by RT-qPCR using a TaqMan probe-based SARS-CoV-2 Nucleic Acid Detection Kit (PerkinElmer, USA) for the presence of SARS-CoV-2 N (via the FAM fluorophore) and Orf1ab (via the ROX fluorophore) genes with 45 cycles on a CFX-96 qPCR machine (Bio-Rad, USA). MS2 phage extraction positive control (PerkinElmer, USA) was added to samples after bead-beating during the RNA extraction process, and sample inhibition was measured according to MS2 detection via the VIC fluorophore (29). Genome equivalents (or viral copy numbers) were estimated using the average Cq values from two technical replicates and calculated based on the mean intercepts and slope for N gene (intercept = 43.6, slope = −3.47) and Orf1ab gene (intercept = 42.5, slope = −3.38) generated from standard curves using five dilutions of the Twist synthetic SARS-CoV2-RNA control 1 (GenBank ID: MT007544.1, Cat no: 102019) (29). Samples identified as RT-qPCR positive for SARS-CoV-2 at one or both loci were subjected to hemi-nested PCR and amplicon sequencing analysis.

### Hemi-nested PCR and amplicon sequencing

Samples identified as SARS-CoV-2 positive by RT-qPCR (i.e., by N and/or Orf 1ab genes) were subjected to a two-stage hemi-nested PCR. In the hemi-nested PCR, a primary RT-PCR (20 µL) was performed using 5 µL of RNA targeting the most variable and informative NTD and RBM regions of the Spike gene using specific primers (0.4 µM each) (Table S3) and the LunaScript Universal One-Step RT-qPCR Kit (NEB). Primary RT-PCR amplification was performed as follows: 55°C (10 min) + 95°C (3 min) + [95°C (30 s) + 60°C (30 s)] × 45 cycles on a T100 thermal cycler (Bio-Rad, USA). A secondary hemi-nested PCR (20 µL) was performed using 2.5 µL of the primary RT-PCR as a template with the same forward primer and a hemi-nested reverse primer for both the RBM and NTD (0.4 µM each) (Table S3). Hemi-nested PCR was performed as follows: 95°C (3 min) + [95°C (30 s) + 60°C (30 s)] × 25 cycles on a T100 thermal cycler (Bio-Rad, USA). Excess primers and nucleotides were removed from the hemi-nested PCR products using ExoSAP-IT (ThermoFisher), according to the manufacturer’s instructions.

An overhang (OH) PCR, adapted from references (30, 31) was then performed using two sets of gene-specific primers, each containing a unique 5′ OH sequence (OH1 or OH2) (Table S3). OH PCR was assembled using GoTaq (2×) Mastermix (Promega) with 1 µL of hemi-nested product as a template and performed as follows: 95°C (3 min) + [95°C (15 s) + 60°C (30 s) + 72°C (30 s)] × 10 cycles + 72°C (7 min). OH1 and OH2 PCR products were purified using NucleoMag NGS beads (Macherey-Nagel) in a 1.5:1 (bead:product) ratio, as per the manufacturer’s instructions. An indexing PCR was assembled using GoTaq (2×) Mastermix together with Illumina-compatible primers and performed as follows: 95°C (3 min) + [95°C (15 s) + 60°C (30 s) + 72°C (30 s)] × 16 cycles + 72°C (7 min). As described above, the indexed products were pooled together and purified using NucleoMag NGS beads. The final library was evaluated using a 4200 TapeStation automated electrophoresis system (Agilent) and quantified using the Qubit HS dsDNA assay (Invitrogen). The library was diluted and prepared for sequencing on an Illumina MiniSeq using Mid-Output Kits (~8 M reads/kit), according to the manufacturer’s instructions, to generate 281 bp (fwd) and 21 bp (rev) paired-end reads.

### Data analysis and variant calling

Reads were analyzed using a custom pipeline (see Data Availability). Briefly, raw reads were demultiplexed with Cutadapt (32), then adapter and primer sequences and low-quality bases were trimmed with Trimmomatic (33). For SNP and indel detection, the reads were first aligned to the SARS-CoV-2 genome with BWA-MEM2 (34). The resulting BAM files were sorted and indexed with SAMtools (35), and low coverage regions were masked with BEDTools (35). Indel quality information was added, and after re-sorting and indexing, variants were called and written to VCF with LoFreq (36). SNPs and indels with an allele frequency ≥ 0.02 and depth ≥ 400 were extracted from each VCF with GATK VariantsToTable (37) and renamed from nucleotide to codon nomenclature using a custom reference table Table S1). The data were then summarized, and SARS-CoV-2 variants were typed according to the presence of signature mutations (Table 5) using R version 4.2.3 (38).

## RESULTS

Of the 411 wastewater samples collected from 15 November to 21 December 2021 and tested for SARS-CoV-2 by RT-qPCR for N and Orf1ab genes, 92% (*n* = 379) were positive at both diagnostic loci (i.e., N and Orf1ab) with a Cq mean = 34.5; *ca* ~ 654.8 viral equivalents per reaction, whereas 8% (*n* = 32) were positive at only a single locus (i.e., N or Orf1ab) with a Cq mean > 38.6; *ca* ~ 26 viral equivalents per reaction (Table 1; Table S1). By hemi-nested PCR amplification of the highly variable and informative regions (i.e., NTD and RBM) of the SARS-CoV-2 spike gene followed by amplicon sequencing of the hemi-nested PCR amplicons, we could sequence and type the variants in 317 (77%) of the 411 RT-qPCR-positive samples. This included 244 out of 288 samples (~85%) that were positive at both diagnostic loci with a Cq mean ≤ 36 and 62 out of 91 samples (~68%) with a Cq mean > 36 at both diagnostic loci (Tables 2 and 3). However, only 11 out of 32 samples (34%) that were positive at a single loci (Cq mean > 36) were successfully typed for the variants (Tables 2 and 3).

**TABLE 1:** Summary of SARS-CoV-2 diagnostic RT-qPCR results of samples analyzed in this study

SARS-CoV-2 RT-qPCR (N and Orf1ab)	Loci	No. samples	% samples	Cq (mean)	Cq (range)	Estimated copies/reaction (mean)	Estimated copies/sampler (mean)
Dual loci positive	N	379	92.2	34.5	30.0–39.5	654.8	6,548
	Orf1ab			34.5	29.6–40.9		
Single loci positive	N	18	4.4	38.3	37.2–39.8	37.32	373
	Orf1ab	14	3.4	38.8	37.1–41.9	15.18	152
	**Total**	**411**	**100.00**				

**TABLE 2:** Comparison of amplicon sequencing performance in samples with higher (RT-qPCR Cq ≤ 36) and lower (Cq > 36) SARS-CoV-2 gene copy numbers.

Condition	SARS-CoV-2 RT-qPCR	No. samples	No. samples successfully sequenced (NTD and/or RBM)	% samples successfully sequenced
Cq ≤ 36	Dual loci positive (N and Orf1ab)	288	244	84.7
	Single loci positive (N or Orf1ab)	0	N/A^*a*^	N/A
	**Total**	**288**	**244**	**Avg 84.7**
Cq > 36	Dual loci positive (N and Orf1ab)	91	62	68.1
	Single loci positive (N or Orf1ab)	32	11	34.4
	**Total**	**123**	**73**	**Avg 51.2**

^*a*^ N/A, not applicable.

**TABLE 3:** Comparison of amplicon sequencing performance in dual and single loci positive samples

SARS-CoV-2 RT-qPCR (N and Orf1ab)	No. samples	No. samples successfully sequenced (NTD and/or RBM)	% samples successfully sequenced
Dual loci positive (N and Orf1ab)	379	306	80.7
Single loci positive (N or Orf1ab)	32	11	34.4
**Total**	**411**	**317**	**Avg 57.5**

In 13% (*n* = 54) of the samples analyzed, either the NTD or RBM failed to sequence successfully (Table 4). However, there was sufficient information to type the SARS-CoV-2 variants in these samples due to the presence of multiple lineage-defining mutations present in the other region (Table S2). Of the 54 samples, the majority (*n* = 47) were RT-qPCR positive at both loci (Cq mean = 35.2; *ca* ~ 343.7 viral equivalents per reaction), whereas the other seven samples were single-loci positive (Cq mean = 38.9; ca ~ 21.5 viral equivalents per reaction) (Table 4). The reason that only one region was sequenced successfully likely reflects the stochastic nature of SARS-CoV-2 RNA in wastewater, keeping in mind that it is often fragmented or degraded and therefore may not provide coverage of the entire genome. This effect is only exacerbated in samples with high Cq and low viral load. The duplex assay may have contributed to reducing assay performance to some extent. However, targeting multiple genomic loci may increase sensitivity for detecting variants at low abundance given that, for example, Omicron was detected in the RBM and not the NTD (e.g., #36219 and #37709), and vice versa (e.g., #36823 and #38333) (Table S2).

**TABLE 4:** Breakdown of amplicon sequencing results by detection and RT-qPCR result

Amplicon sequencing result	SARS-CoV-2 RT-qPCR (N and Orf1ab)	No. samples	% samples	Cq (mean)	Cq (range)	Estimated copies/reaction (mean)	Estimated copies/sampler (mean)
Omicron and Delta variant of concern (VOC) detected (NTD and RBM amplicons sequenced)	Dual loci positive (N and Orf1ab)	21	5.1	34.4	31.8–38.2	596	5,960
	Single loci positive (N or Orf1ab)	0	0.0				
	**Subtotal**	**21**	**5.1**				
Delta VOC detected (NTD and RBM amplicons sequenced)	Dual loci positive (N and Orf1ab)	238	57.9	34.2	29.8–39.8	788.6	7,886
	Single loci positive (N or Orf1ab)	4	1.0	38.5	38.0–39.3	16.5	165
	**Subtotal**	**242**	**58.9**				
Unable to sequence	Dual loci positive (N and Orf1ab)	73	17.8	35.3	30.5–38.3	436	4,360
	Single loci positive (N or Orf1ab)	21	5.1	38.4	37.2–40.2	31.7	317
	**Subtotal**	**94**	**22.9**				
VOC detected (only NTD or RBM amplicon sequenced)	Dual loci positive (N and Orf1ab)	47	11.4	35.2	32.0–38.0	343.7	3,437
	Single loci positive (N or Orf1ab)	7	1.7	38.9	38.1–41.9	21.5	215
	**Subtotal**	**54**	**13.1**				
	**Total**	**411**	**100.00**				

Based on our typing methods, wherein variants were evaluated according to their NTD and RBM single nucleotide polymorphism (SNP) profiles (Table 5), the Delta variant (SNPs: T95I, G142D, E156G, Δ157/158, L452R, and T478K) was detected in 296 samples, with a mixture of both Omicron (SNPs: A67V, Δ69/70, T95I, G142D, Δ143/145, K417N, N440K, G446S, S477N, T478K, E484A, Q493R, G496S, Q498R, and N501Y) and Delta variants detected in 21 samples (Table 2). Omicron was not detected in any of the samples collected between 15 November 2021 [approximately 1 week prior to the first global report of Omicron (12, 13)] and 30 November 2021 (*n* = 137). All samples collected during this time contained the Delta variant only (Table S2), consistent with an ongoing, substantive Delta outbreak in Victoria, peaking in October 2021.

**TABLE 5:** Characteristic SNPs in the NTD and RBM amplicon regions associated with SARS-CoV-2 VOC^*a*^^,^^*b*^

	Alpha B.1.1.7	Beta B.1.351	Gamma P.1	Delta AY.X	Omicron BA.1
A67V	^—^	^—^	^—^	^—^	^+^
Δ69/70	^+^	^—^	^—^	^—^	^+^
D80A	^—^	^+^	^—^	^—^	^—^
T95I	^—^	^—^	^—^	^+^	^+^
D138Y	^—^	^—^	^+^	^—^	^—^
G142D	^—^	^—^	^—^	^+^	^+^
Δ144	^+^	^—^	^—^	^—^	^—^
Δ143/145	^—^	^—^	^—^	^—^	^+^
E156G	^—^	^—^	^—^	^+^	^—^
Δ157/158	^—^	^—^	^—^	^+^	^—^
K417N	^—^	^+^	^—^	^—^	^+^
K417T	^—^	^—^	^+^	^—^	^—^
N440K	^—^	^—^	^—^	^—^	^+^
G446S	^—^	^—^	^—^	^—^	^+^
L452R	^—^	^—^	^—^	^+^	^—^
S477N	^—^	^—^	^—^	^—^	^+^
T478K	^—^	^—^	^—^	^+^	^+^
E484A	^—^	^—^	^—^	^—^	^+^
E484K	^—^	^+^	^+^	^—^	^—^
Q493R	^—^	^—^	^—^	^—^	^+^
G496S	^—^	^—^	^—^	^—^	^+^
Q498R	^—^	^—^	^—^	^—^	^+^
N501Y	^+^	^+^	^+^	^—^	^+^

^*a*^ SNP prevalence information was sourced from https://outbreak.info on 8 December 2021. BA.1 sequences are highly in flux; therefore, SNPs associated with this variant are subject to change as more sequences are reported. The table is intended as a reference only and is not exhaustive.

^*b*^ +, SNP observed at >75% prevalence of lineage sequences; —, SNP observed at <75% prevalence of lineage sequences.

Many additional mutations were detected alongside the mutation profiles of Delta and Omicron (e.g., T76I, G72E, G75D, T95A, Y145C, and L441P) (Table S2). These non-characteristic mutations were largely detected in low abundance (i.e., 2%–10%) and are likely indicative of the inherent heterogeneity in circulating variants not always represented in clinical whole-genome sequence data sets, which only report the consensus sequence. These mutations are important to monitor over time, particularly those observed in successive sampling, as they may indicate the emergence of new sub-lineages [see references (39, 40)].

Our wastewater-based epidemiology program detected the first known incursion of the Omicron BA.1 variant into Victoria before any confirmed clinical cases. Twenty-four-hour passive wastewater samples collected between 1 December and 2 December 2021 (sample #36213) from Victoria’s major international airport, Tullamarine Airport, and a larger catchment sampling site incorporating Tullamarine Airport (sample #36219) both tested positive for SARS-CoV-2 on 5 December 2021 as part of the state’s routine SARS-CoV-2 wastewater surveillance program. On 6 December, these samples were sent for variant typing, and the positive detection of Omicron BA.1 in each sample was reported back to the Victorian Department of Health on 8 December 2021. Omicron BA.1 was later detected from consecutive samples (#36811 and #37009) collected on 7–8 December and 8–9 December from a large central Melbourne catchment, “Melbourne Main” (Table S2), where several quarantine hotel facilities were located. These quarantine facilities included those accommodating passengers testing positive for SARS-CoV-2 by routine PCR screening upon arrival at Tullamarine Airport.

The unexpected wastewater detections were escalated to Victoria’s Chief Health Officer on 8 December 2021. As a result, on 9 December 2021, the Victorian DH requested that the public health laboratory responsible for clinical genomic sequencing of SARS-CoV-2 cases in Victoria (1) review all PCR screening results for travelers arriving at Tullamarine Airport on or after 1 December 2021 and (2) fast-track their genomic sequencing. This review identified one SARS-CoV-2-positive arrival at Tullamarine Airport on 1 December 2021, classified as a suspected Omicron BA.1 infection based on S-gene PCR dropout (41). The Victorian Chief Health Officer then issued an urgent press release and related health advice on 9 December 2021 and initiated additional contact tracing and isolation measures for those having suspected contact with the Omicron case. The suspected Omicron BA.1 infection classification was subsequently confirmed by genomic sequencing. Thus, within 3–4 days of case arrival and before sequence results from clinical samples, WBE demonstrated repeat detections of the incursion of the BA.1 variant into the Victorian population and supported a case review and public health response following those initial detections.

## DISCUSSION

In Victoria, the SARS-CoV-2 WBE program, active since August 2020, has been used adaptively to inform the COVID-19 public health response across a wide range of urban and regional areas and in multiple localized settings (42). Until late 2021, the program focused on viral detection amidst a background of low community-case transmission, with the primary aim of assisting early detection of new community infections and containment to limit onward transmission. However, as community transmission increased and wastewater detections of SARS-CoV-2 became the norm rather than the exception, the program shifted toward detecting and typing SARS-CoV-2 variants and quantitative trends.

Here, we have shown how targeted and highly sensitive amplicon sequencing of wastewater can be used for early detection of emerging SARS-CoV-2 variants as part of an adaptive collaborative wastewater surveillance program, which includes international airports. Our method identified the first known incursion of BA.1 into Victoria amidst a dominant background of the Delta variant. The detections of BA.1 align temporally and spatially with a subsequently confirmed Omicron BA.1 case. This directly supported an urgent public health response aimed at slowing the spread of the Omicron variant and optimizing health system preparedness for the first Omicron wave. This strategy for using variant data from wastewater surveillance to identify incursion of novel SARS-CoV-2 variants has since been incorporated into the Australian Government Department of Health’s national strategy for SARS-CoV-2 surveillance in wastewater (43).

The sensitivity of our method was demonstrated by the detection of BA.1 mutation profiles amidst a high background of Delta and the detection of additional non-characteristic mutations that may represent viral sub-lineage evolution. Our duplex method provides coverage of both RBM and NTD regions and employs a novel reverse-overhang sequencing approach that enables sequencing of amplicons up to 562 bp in length and can be run on the MiniSeq platform, which is cost-effective and has a short run time (~17 h). Studies have shown the emergence of antibody escape mutations in both the RBD and the NTD, suggesting that although the RBD is immunodominant, mutations in either domain play a substantial role in antigenicity (26). Our duplex amplicon sequencing assay, which targets both RBM and the NTD, is therefore useful for monitoring emerging strains with a potentially higher capability for immune evasion. Other targeted amplicon sequencing methods have been reported for wastewater, targeting the RBM alone (19) or with the NTD and S1/S2 subunit split region (39, 40). However, to our knowledge, ours is the first study to comprehensively assess these methods' sensitivity, performance, and timeliness in a comparatively low case-load setting and specifically within an operationalized public health program.

### Conclusions

WBE at ports of entry and geographic areas using passive sampling combined with targeted amplicon sequencing provides high population coverage and sensitive and early detection of low abundance variants enabling a timely public health response. This is used as a complementary surveillance tool alongside clinical genomic surveillance providing geospatial and temporal information complementing the individual data required for contact tracing and further viral, demographic, and clinical characterization.

Our targeted amplicon sequencing method is rapid, cost-effective, and sensitive. Importantly, because the amplicon primers target conserved regions that flank highly polymorphic loci, they should be less prone to dropout and remain suitable for new variants. Nonetheless, updates to the primers may be required over time to incorporate new variants or additional informative loci or to extend the boundaries of the existing regions in response to changes in SARS-CoV-2 genetics. This approach also has relevance for low and middle-income settings, many of which are currently under-represented in global surveillance data or may be adapted to other viruses [e.g., influenza (44)] or pathogens where typing a highly polymorphic sequence is desirable. Further evaluation of these methods, including more recent samples incorporating the many novel Omicron and recombinant sublineages and global polyclonalism of SARS-CoV-2 that has emerged since mid-2022, is also needed.

## Data Availability

Illumina MiniSeq data are available under NCBI BioProject PRJNA992940. Analysis scripts are available at https://github.com/Jex-Lab/WW_surveillance_of_SARS-CoV-2

## SUPPLEMENTAL MATERIAL

Supplemental tables aem.01497-23-s0001.xlsx Tables S1 to S3.

ASM does not own the copyrights to Supplemental Material that may be linked to, or accessed through, an article. The authors have granted ASM a non-exclusive, world-wide license to publish the Supplemental Material files. Please contact the corresponding author directly for reuse.
